# How Academic Biologists and Physicists View Science Outreach

**DOI:** 10.1371/journal.pone.0036240

**Published:** 2012-05-09

**Authors:** Elaine Howard Ecklund, Sarah A. James, Anne E. Lincoln

**Affiliations:** 1 Department of Sociology, Rice University, Houston, Texas, United States of America; 2 Department of Sociology, Rice University, Houston, Texas, United States of America; 3 Department of Sociology, Southern Methodist University, Dallas, Texas, United States of America; Northwestern University, United States of America

## Abstract

Scholars and pundits alike argue that U.S. scientists could do more to reach out to the general public. Yet, to date, there have been few systematic studies that examine how scientists understand the barriers that impede such outreach. Through analysis of 97 semi-structured interviews with academic biologists and physicists at top research universities in the United States, we classify the type and target audiences of scientists’ outreach activities. Finally, we explore the narratives academic scientists have about outreach and its reception in the academy, in particular what they perceive as impediments to these activities. We find that scientists’ outreach activities are stratified by gender and that university and disciplinary rewards as well as scientists’ perceptions of their own skills have an impact on science outreach. Research contributions and recommendations for university policy follow.

## Introduction

If science is going to fully serve its societal mission in the future, we need to both encourage and equip the next generation of scientists to effectively engage with the broader society in which we work and live. – Alan Leshner.

As the United States continues to fall behind other countries in math and science performance [1], Alan Leshner, CEO of the American Association for the Advancement of Science, expresses a sense of urgency about translating science to the broader public. Further, the mid-1990s implementation of a National Science Foundation grant application Broader Impacts Criterion mandates outreach as part of the granting process for the nation’s researchers [2], stating that those seeking funding must provide a description of how a proposed research project will affect the broader society via teaching, inclusion of underrepresented groups, the creation of outreach relationships, public discussion of research findings, and general social benefits of the project (See http://www.nsf.gov/pubs/gpg/broaderimpacts.pdf Accessed 4/1112). By attaching such a directive to research funding, the NSF compels scientists to engage in such outreach, underscoring its importance to major science funding bodies.

These are signs that scientists have renewed their interest in outreach efforts, which Burns, O’Connor, and Stocklmayer (2003) define as any activity in which scientists translate their research or broader scientific concepts to those outside of the academy. Here we are most interested in the outreach that academic scientists say they undertake rather than examining the impact of scientists’ efforts to transmit scientific knowledge to the public. Previous research shows that half of academic scientists are engaged in some type of outreach [3], [4], though 5 percent of the most active public scientists do half of all outreach [4].

However, existing research on this important topic is limited. The small body of existing scholarship on science outreach reveals that stage of career is a salient factor in outreach participation, with senior scientists more likely to take part in one-time-only opportunities, like being a guest on a TV or radio program and junior scholars more likely to engage in primary and secondary education outreach [4]. The broadest body of literature deals with the factors that prevent scientists from more extensive engagement in outreach activities, with the most commonly cited barriers as time, funding, knowledge, training, and institutional disincentive. There is also a widely perceived “Sagan Effect” or a professional stigma attached to spending too much time translating one’s research to the broader public [5]. Scientists who think their colleagues do little are less likely to display an interest in outreach work themselves [6], even though researchers find that in terms of tenure and promotion [4] outreach activity has a small, positive effect on the science career.

Myriad factors play a role in scientists’ perceived ability to engage in outreach. For more than half of all scientists, a lack of time is the most insurmountable barrier to doing more outreach [7], [8], [9], and perceived time constraints are associated with a more negative impression of doing outreach activities [6]. This time pressure may be compounded by inadequate distribution of knowledge about available outreach opportunities, forcing scientists to expend considerable effort to create or locate existing outreach options [1], [7]. Some researchers argue that scientists feel they do not have the necessary skills to share their research [9]. Scientists often perceive themselves as having poor personal communication skills [1] and have little confidence in their own abilities to do outreach [6], leading scientists to think they might actually hurt the public’s perception of science if they engage in outreach activities.

Lack of encouragement at the institutional level is another common impediment to the participation of scientists in outreach activities [8], [9]. Little support of such work from departments, mentors, and advisors is a salient barrier for both graduate students and faculty members [7]. Additionally, a widespread conception among academics is that dissemination of research findings beyond peer-reviewed journals is “dumbed-down” [9] science and thus not undertaken by the most talented of researchers [10]. Therefore, little institutional assistance or approval is given for the creation of outreach programs or involvement in outreach opportunities [8].

Research on the popularization of outreach activities for U.S. scientists is lacking (For one exception to this see a report by the National Science Foundation which shows that 42 percent of scientists engaged in no public outreach. Among others, scientists gave the reasons of not having time and not valuing outreach. See http://pus.sagepub.com/content/20/1/3.full.pdf, accessed 4/11/12).

### Gaps in Research

In general there is more comprehensive investigation of the public’s understanding of science and perception of science outreach [11], [12], [13] than investigation of perceptions of outreach among the scientists with whom the public interacts. To date, there have been no nationally representative studies to determine which scientists are engaged in outreach or what types of outreach they do. Investigation of these questions has most often been programmatically driven, where scientists involved in a particular activity are queried about the frequency of their outreach participation and motivation for participation. While there is some research on perceived barriers to outreach, such barriers are not explored in depth, and little research [8] has occurred after the implementation of the Broader Impacts Criterion for evaluating National Science Foundation grant applications. Finally, we know little about the views of scientists’ outreach efforts across a broad variety of institutions and disciplines in the United States, other than that most scientists portray science outreach in a negative light [14]. And there is lack of knowledge about how scientists at elite academic institutions, in particular, view these activities and about the attitudes of their institutions toward such work, despite the fact that some scientists at elite research universities are leaders in their disciplines, more likely to set the tone for science outreach initiatives nationwide. In short, the onus of science outreach work is put on scientists’ shoulders, yet we know little about what scientists themselves think about issues of outreach, how it ought to be done, and what strategies could be most effective in creating better outreach efforts.

To fill these important gaps, we conducted semi-structured interviews with a random sample of academic scientists at elite universities in the United States, classifying respondents’ outreach activities in relationship to their target audiences. We investigate whether scientists at elite research universities engage in science outreach at all and, if so, what types of projects they undertake. Finally, we also ask what impediments scientists face when attempting to engage in outreach efforts and what strategies scientists believe the scientific community could be using to facilitate such efforts.

The sample for this study was randomly selected from a larger study of Perceptions of Women in Academic Science (PWAS), which included a survey and in-depth interviews with scientists housed in the top twenty graduate programs in biology and physics –two core science disciplines– in the United States. Both survey and interview questions focused on scientists’ perceptions of challenges they faced throughout their careers. During just the interview portion of the study, respondents were also asked about involvement in science outreach efforts, though this topic was not included in the survey. An initial survey sample of 3,455 scientists was chosen randomly from among all graduate students, postdoctoral fellows, and tenure-track and tenured faculty members in departments with the top 20 graduate programs in all subfields of physics, astronomy, and biology as ranked by the National Research Council (1995) and corroborated by the more recent U.S. News & World Report rankings (2008). The survey achieved a 72 percent response. Following completion of the survey in February 2009, we conducted semi-structured qualitative interviews with a smaller random subsample of those who completed the survey, resulting in 150 interview respondents. Ninety-seven of these respondents were asked questions about their perceptions of science outreach and their specific outreach activities, including the following:

1. I wonder if you are involved in any work aimed at translating science to individuals outside the academy or the scientific community. Could you tell me a little about these efforts?
2. Do you think scientists in general are doing a good enough job at translating science to broader communities? Why or why not?
3. [If no to above] How could they be doing a better job?

The interviews were entirely transcribed. A coding scheme was developed, and all interviews were coded. Inter-coder reliability checks were conducted, in which two coders coded the same interview and their work was checked for consistency. The inter-coder reliability check had a reliability statistic of. 90.

## Results

### Demographic Correlates of Science Outreach Efforts

Overall, 58 percent of the respondents are involved in some type of science outreach pursuit. Though biologists and physicists are equally involved in such efforts (χ^2^ = 2.66, df = 1, p = 0.103), women are markedly more involved in outreach work than men (72 percent versus 43 percent, χ^2^ = 8.59, df = 1, p = 0.003), a finding that holds within each discipline. The difference is larger in biology, where 69 percent of women but only 32 percent of men do outreach work. In physics, 76 percent of women are engaged in some type of science outreach work when compared to 58 percent of men. These gender differences are significant (χ^2^ = 11.91, df = 3, p = 0.008). Correspondingly, it is important to note that while women in physics are more likely than men in physics to do outreach work, the overall numbers of women in the discipline are very small. (Less than 7 percent of full professors in physics at these universities are women.) Between the two largest racial groups, whites are more likely to take on science outreach work (63 percent) than are Asians (39 percent), but there are too few scholars of other racial groups to extrapolate meaningful participation rates and ultimately these racial differences are not statistically significant (χ^2^ = 2.59, df = 2, p = 0.271). We also find that 54 percent of graduate students, scientists at the beginning of their careers, are involved in outreach while the proportion drops to one-third among postdoctoral fellows. There is no meaningful difference, however, in the proportion of tenure-track faculty (71 percent) and tenured faculty (69 percent) who take on science outreach work, and overall, the differences in participation rate between groups is not significant (χ^2^ = 5.77, df = 4, p = 0.216).

Surprisingly, there is no difference in science outreach efforts between those who have children and those who do not (66 percent vs. 52 percent, χ^2^ = 1.93, df = 1, p = 0.166). Further, there is no significant difference between scientists with children under age 5 (i.e. not school aged) who do outreach, and those with children between ages 5 and 10 (elementary school aged) who do outreach (63 percent versus 64 percent, χ^2^ = 0.122, df = 2, p = 0.941). Rather, the relationship between science outreach involvement and parental status is split along gender lines. Eighty-one percent of women with children do outreach, as compared to 66 percent of women without children. For men, 50 percent with children do science outreach while 37 percent of men without children are involved. By discipline, biologists with or without children have comparable levels of outreach participation (54 percent and 48 percent, respectively). Physicists have a much sharper distinction; 82 percent of physicists with children are engaged in outreach as opposed to just 56 percent of those without children. We thought that scientists might be predisposed to doing outreach involving their own children’s schools, but this does not seem to be the case as only three respondents mentioned doing so, all of which involve their children’s classes at school.

### Types of Science Outreach

A plurality of scientists who are involved in science outreach are engaged in some type of outreach that involves school-aged children (32 percent of respondents). The majority of these efforts focus on giving presentations to either elementary school or high school students. Bringing students into their own labs is another way that scientists engage in science outreach efforts although these tend to be undergraduates who are involved in their labs. For example, only a handful (4 percent) of those involved in outreach have high school students working in their labs. A few respondents are involved in classrooms in another way, by working with primary and secondary teachers to develop better practices for teaching science to a younger audience (3 percent). About 21 percent of respondents engage in science outreach efforts that target the general public, via activities such as giving public lectures or writing science books for non-specialists. Another 6 percent aim their outreach at another specific group, such as those in the private investment sector.

### Barriers and Proposed Solutions

In scientists’ own words, science outreach is a bleak prospect with limited room for improvement. Seventy-four percent of respondents list one or more significant impediments to their ability to do science outreach, yet less than half have concrete ideas for how science outreach could be improved. For the less than 10 percent of respondents who want to dedicate their career to science outreach, most report facing significant disapproval of this choice while completing their academic training during graduate school or a postdoctoral fellowship. A graduate student in physics (Phys41M, conducted 5/24/10) describes his experience as a scientist with the desire to teach at a community college, which he sees as a career devoted to outreach because his work will be centered on training future science teachers:

The best way you can do it is to keep your mouth shut and keep going until you finish. If [mentors] realize that you don’t want to become them [university professors] eventually, well, then they’ll basically not give you enough to work with - enough resources or time or investment on their part for you to finish your PhD. … It’s medieval.

The barriers to science outreach are generally attributed to one or more of the three elements that shape science outreach: scientists, the academy, and the public.

### Scientists

Thirty-seven percent of respondents place the blame for poor science outreach efforts on scientists themselves. Twenty-nine percent of all respondents say that scientists are poor interpersonal communicators or that nonscientists perceive them to be uniformly inept at communication, regardless of their actual abilities. A male biologist, who is an assistant professor (Bio4M, conducted 6/20/09) said: “I’m not sure you want most of the people that I know here to go out and try to talk to the public. They’re [the public] gonna say ‘stop spending my tax dollars on this person!’” Yet only two respondents (2 percent of the sample) suggested training scientists how to be better communicators.

Another 5 percent say that scientists are not interested in doing outreach because they do not see it as part of their role as a scientist; these scientists believe that it is not their job to interpret their work for a broader audience. As a solution, about 15 percent think there is a need for non-scientists to organize scientists’ outreach efforts. Examples given include a university outreach organizer or an outside outreach organization. Many believe that scientists are simply not the appropriate people to teach those outside the scientific community about science. A male physicist, who is an associate professor (Phys38M, conducted 5/13/10), expressed the lack of agreement among scientists about the right way to approach communication with those outside the academic science community:

I guess it’s unclear whether the scientists themselves are the right people to do the communicating or whether an intermediary is what’s most useful. So, you know, my guess is that most scientists like the idea but some hold it high and others have sort of conflicted feelings about whether one should be spending one’s own time doing something versus just doing what you’re good at and communicating it to other people who are very good at communicating it to people at large.

The debate centers on whether it is more important for the public to receive information directly from a scientist who is doing academic research or from a third party who is informed by the academic scientist and who may be a more effective communicator than the scientist.

### The Academy

About 31 percent of scientists interviewed think the academy is at fault for poor science outreach. According to these scientists, in a research university system that seems to value research productivity over all else, institutions do not train scientists to do outreach. Prioritizing research and publications leaves scientists feeling that they have little time to engage in activities that are not directly connected to their academic pursuits. And a lack of outreach program infrastructure and few easy-to-locate opportunities make actually following through with outreach efforts both time and labor intensive for scientists.

Scientists also perceive that they are rewarded little for science outreach work, especially in the tenure process. A theme voiced by 19 percent of respondents in their suggestions for improving outreach activities is that scientists need recognition and respect in the academy for their outreach efforts if they are to pursue these activities. Some respondents suggested that the academy as a whole needs to reevaluate its values if it wants to continue to receive funding from an increasingly skeptical public and private investment sector. A female, associate professor of physics, (Phys24F, conducted 3/16/10), highlighted the financial necessity of convincing the public that academic science is meaningful:

During the Cold War era, physics really benefited from the umbrella of money that came in … [and were] not responsible enough about communicating why the government should fund basic research and why it’s good for somebody who otherwise isn’t very interested in science. … In physics we’ll have to do a better job of describing to the public why it’s important to put money into basic research even when the country’s in crisis.

This particular example shows that there was some difference between physicists and biologists in the necessity of outreach to their discipline, with physicists seeing convincing the public of the legitimacy of their research as perhaps central to research funding for physics.

Some respondents not only view outreach as a misuse of time that could be better spent on research, but believe it to even be detrimental to career advancement or prestige. A biologist, who is an assistant professor (Bio45F, conducted 4/23/10), described her colleagues’ views of outreach as overwhelmingly negative:

I think that people look down on the popularizer, and I think that’s a real big mistake personally. I think that popularizers are really important, and being able to explain stuff to the public is really important. And so I don’t think we should, you know, denigrate those people at all [*laughs*].

A negative view of those who work to spread their scientific expertise beyond the academy may be tied to the “Sagan effect,” where individuals who are more accessible to the public are thought to do less rigorous scientific research [4]. As the quote above suggests, some scientists think that too much time spent on outreach will cause others to perceive them as “popularizers” like Sagan. Views on the status of the popular scientist are mixed, because even as some respondents denounced Sagan, several respondents cited the need for a new figurehead who could lead nationwide outreach efforts. A male physics professor, (Phys15M, conducted 2/4/10), described this potential leader as “someone like a Nobel laureate” – pointing to the importance of selecting a figurehead who is well respected by both the scientific community and the general public.

### The Public

Roughly a quarter of respondents suggest that a central barrier to effective science outreach is the public itself. Of those who mention characteristics of the public as an impediment, 70 percent express a perception of public ignorance, while 30 percent blame a disinterest in science. Scientists have the perception that a widespread lack of scientific knowledge among the general public is a difficulty in communicating advanced scientific discoveries beyond the borders of the academic science community. This view fits the deficit model of science communication, where scientists view their role in outreach as mainly to fill a void in knowledge among members of the general public. A biology graduate student (Bio58F, conducted 7/12/10) expressed the downside of this approach, saying that she thinks the public views scientists as “snobby intellectuals making a judgment on high.” This statement also reflects scientists’ frustration with a public that does not appreciate science broadly as well as the public’s sense of detachment from academic science in particular.

However, some scientists feel widespread disinterest in science and mistrust of scientists is a more pressing issue than a lack of science knowledge among the public. They believe that the public is simply apathetic or even opposed to learning about science and the scientific process, meaning that outreach efforts will have little impact. A biology professor (Bio11F, conducted 7/15/09), explained the barriers she sees to approaching such an audience:

There is an increasingly large sector of our population that doesn’t want to hear about science, is afraid of technology, is afraid of scientific knowledge, doesn’t want their children to learn science, is actively working to make sure their children don’t learn science. … When somebody doesn’t believe what you are doing is true or has any value, then trying to explain to them what you are doing, you’re starting from this cultural foundation that is a complete disconnect.

Respondents expressed concern about both public ignorance of and disinterest in science, but felt that only issues of public ignorance could be remedied. Scientists argued that encouraging the public to be excited about science might even be a hopeless prospect. With visions of remedying at least some of the scientific illiteracy that they see as paralyzing the public, however, 8 percent of respondents reiterated the necessity of improving pre-college science education. They place the burden of this work not on the public school system or individual campuses, but instead on scientist themselves, who must make more of an effort to connect with school-aged students. A physics postdoctoral fellow, (Phys12M, conducted 2/3/10), suggested the integration of university physicists into primary and secondary educational settings. According to him, “maybe one of the best things would be to embed some scientists in a grade school or junior high a few times a week or a few times a month. It seems to me that would be a pretty effective way to reach a lot of people.” Unlike some of his physicist colleagues, this scientist thinks the public’s attitude toward–and acceptance of–science would improve if more individuals in the public (starting in grade school) had the opportunity to simply interact with scientists.

Additionally, 10 percent of respondents mention technical language barriers. The vocabulary that scientists are accustomed to using to describe their work is largely unfamiliar to the layperson and, as a biology graduate student (Bio2M, conducted 6/9/09) explained, in his sense of things it is important not only to address unfamiliar vocabulary, but also to make sure that the way the concept is described is accessible to the audience: “This sounds mean, but you dumb it down a little bit. And I don’t mean to make that sound bad, but necessarily so.” The overall consensus among scientists is that both scientists and the public are to blame for poor science communication.

## Discussion

A central finding of this research is that, among biologists and physicists at top research universities included in this study, women are much more involved in outreach than men. One interpretation of this finding is that, as the number of women in academic science increases, science outreach may increase. A corresponding interpretation is that scientists may have the perception that outreach is a more feminine, care-oriented task, which may further decrease the legitimacy of this pursuit. And unless science outreach efforts increase in legitimacy at top research universities the academic careers of the women who engage in outreach work may actually be hindered.

Also important, these scientists perceive significant barriers to outreach at an individual level, within their institutions, and from the general public. And yet, though they think their departments and universities value research productivity over all else, these academic scientists still engage in outreach activities, even though they mention significant barriers to such engagement. Almost three quarters of respondents list one or more factors that limit their outreach activities. Among these, scientists view their peers as mediocre communicators, whose personal styles cannot be improved, a perception that has significant implications for the provision of science outreach. And a significant minority of scientists are concerned about what they see as the American public’s general ignorance of science, mistrust of scientists, and disinterest in scientific topics.

According to our respondents, widespread change in attitude towards science outreach is difficult–if not impossible–to achieve. Even more challenging to modify are scientists’ perceptions of their role as academics, the priorities of the academy, and the public. Outreach may be seen as outside of the responsibilities of the university scientist, an understanding tied in large part to institutional norms at top research universities that value research productivity over other types of contributions [15]. Adherence to these norms limits the time and ability of scientists to take on other projects and even creates disincentives for participation in outreach–often in the form of disapproval by mentors or department heads. It is likely that this negative regard for outreach work may be tied to a “Sagan effect,” such that a scientist’s research quality is thought to be inversely proportional to the amount of outreach work she does. In short, scientists who popularize or make science too accessible are suspect by their research community [4]. Such efforts could be better recognized at the department and university levels, with some suggesting that these efforts should count towards tenure. Leadership at the departmental level not only legitimizes outreach efforts but, in this case, even makes them normative. And making outreach work seem normal is a sign that department and university leaders are reassessing their priorities.
